# Impact of communication strategies to increase knowledge, acceptability, and uptake of a new Woman’s Condom in urban Lusaka, Zambia: study protocol for a randomized controlled trial

**DOI:** 10.1186/s13063-016-1681-x

**Published:** 2016-12-13

**Authors:** Jessie Pinchoff, Rachna Nag Chowdhuri, Noah Taruberekera, Thoai D. Ngo

**Affiliations:** 1 Research & Knowledge Management Department, Innovations for Poverty Action, 101 Whitney Ave, New Haven, CT 06510 USA; 2 Innovations for Poverty Action Zambia, 26 Mwambula Road, Jesmondine, Lusaka, Zambia; 3 Population Services International, 8 Hillside Road, 2nd Floor, Block B, Metropolitan Park, Parktown, Johannesburg, South Africa; 4 Poverty, Gender and Youth Program, Population Council, One Dag Hammarskjold Plaza, New York, NY 10017 USA

**Keywords:** Family planning, Female condom, Contraception, Zambia, Impact evaluation, Randomized controlled trial

## Abstract

**Background:**

Globally, 220 million women experience an unmet need for family planning. A newly designed female condom, the Woman’s Condom (WC), has been developed featuring an improved design. It is the first dual-protection, female-initiated contraceptive that is a premium, higher price point product. However, market availability alone will not increase uptake. In February 2016 the WC will be distributed with a strong media campaign and interpersonal communication (IPC) outreach intervention. The impact of these on knowledge, acceptability, and use of the WC will be measured.

**Methods/design:**

A baseline survey of 2314 randomly selected 18- to 24-year-old sexually active men and women has been conducted. The WC and mass media will be introduced throughout 40 urban wards in and surrounding Lusaka, Zambia. The baseline survey will serve as a quasi-control arm to determine the impact of introducing the WC with mass media. Half of the wards will be randomly allocated to additionally receive the IPC intervention. A single-blind randomized controlled trial will determine the impact of the IPC intervention on knowledge, uptake, and use of the WC. After one year, another 2314 individuals will be randomly selected to participate in the endline survey.

We hypothesize that (1) the distribution and media campaign of the WC will increase overall condom use in selected urban wards, and specifically use of the WC; (2) the IPC intervention will significantly impact knowledge, acceptability, and use of the WC.

The primary outcome measures are use of the WC, use of any condom, and willingness to use the WC. Secondary outcomes include measures of knowledge, acceptability, and choice of contraception.

Odds ratios will be estimated to measure the effect of the intervention on the outcomes with 95% confidence intervals. All analyses will be based on the intention-to-treat principle.

**Discussion:**

Increasing uptake of dual prevention measures (such as the WC) may reduce incidence of sexually transmitted infections/HIV and unplanned pregnancies. It is important to ensure young, urban adults have access to new contraceptive methods; and, understanding how mass media and IPC impact contraceptive knowledge, acceptability, and use is critical to reduce unmet need.

**Trial registration:**

AEARCTR-0000899. Registered on 26 October 2015.

**Electronic supplementary material:**

The online version of this article (doi:10.1186/s13063-016-1681-x) contains supplementary material, which is available to authorized users.

## Background

Globally, 220 million women experience an unmet need for family planning (FP) [1], and more than 100 million women cite method-related reasons for non-use of modern contraceptives [2]. There is a need to diversify the contraceptive choices for women who have different needs, particularly in developing countries, where women are at high risk for sexually transmitted infections (STIs) such as HIV and may lack control over sexual and reproductive decisions due to gender inequity [3]. The Expanding Effective Contraceptive Options (EECO) project was designed to support the research, development, and introduction of new contraceptive methods, with the goals of addressing method-related reasons for non-use, expanding contraceptive choice, and increasing access to woman-initiated methods in order to meet the reproductive health needs of women and girls worldwide. However, just having a product available does not mean it will be used; strategies to increase uptake are still necessary and often lack evidence.

Through the EECO project, a third generation, newly designed premium female condom (FC) called the *Woman’s Condom* (WC), developed by the Program for Appropriate Technology in Health (PATH), has been marketed in Zambia under the brand name *Maximum Diva Woman’s Condom* (WC) by the Society for Family Health (SFH), the Zambian subsidiary of Population Services International (PSI). Previous iterations of the FC, such as the Care Condom or FC2, have been underutilized. To date, FCs only represents 0.8% of the total condoms distributed by donor nations [3]. Attempts to introduce FCs worldwide have encountered barriers such as weak distribution channels within national health systems and critiques of the product design and marketing. The new WC product features an improved design and will be coupled with a multipronged marketing campaign that addresses some of these concerns. The marketing strategy is more streamlined, with a clearly defined target population. In piloting, the product has received positive feedback on its design and ease of use, and it will be marketed with a higher price point as a premium product in urban settings.

Zambia is an important context for the introduction of the WC. Coupled with the high unmet need for family planning (27.1%), Zambia has an HIV prevalence of 13.3% among the general population [4], twice as high as that in urban areas. Young women aged 18–24 years bear a disproportionate burden of this epidemic, with an HIV prevalence of 11.2% compared to 7.3% among men in the same age cohort [4]. According to the 2013–2014 Demographic and Health Survey, the majority (75%) of Zambian women reportedly have had sex by age 20, and 41% of women and 46% of men report wanting to delay having a child by at least 2 years [4]. The total desired fertility rate is lower in urban areas, 4.2 children versus 5.1 children in rural areas [4]. Currently, the prevalence of any FC brand use is less than 1% [4]. Expanding access and choices in contraceptives is important in urban Zambia for preventing unwanted pregnancy and STI/HIV infection, and the FC is currently not available.

In order to ensure uptake and use of a new contraceptive method, there must be a proactive and well-planned strategy to integrate it into the mix of contraceptive methods available in a country [5]. The new WC should be viewed within the context of a range of contraceptive choices, and access to more options has been shown to increase overall use of contraception. Studies suggest that different contraceptive methods are used as complements, not replacements [5]. It is unclear if condom users will shift their preference from one condom brand or type to another; this study will explore any shift in preferences after the introduction of the new WC onto the market.

In addition to understanding the impact of the new WC on the condom market, it is also critical to develop a strategy to increase knowledge, acceptability, and uptake of the new WC. During the distribution of the WC and its mass media campaign, this randomized controlled trial (RCT) will measure the impact of a interpersonal communication (IPC) intervention. IPC interventions here are community based small groups led by trained IPC agents that will demonstrate proper WC use and promote conversations between sexual partners in condom negotiation to change contraceptive norms and product acceptability. To date, no rigorous evaluations of IPC interventions that aim to improve uptake of products have been conducted, particularly among a general population of young people. Recent research does suggest that exposure to IPC is associated with knowledge acquisition and acceptability of sensitive topics such as condom use [6]; however, there is a lack of causality.

This study will measure the impact of the new, premium WC on the condom market and how it impacts user contraceptive preferences and brand selection. This study will also implement an RCT designed to measure the added impact of a community-based IPC intervention on improving WC (and overall condom) knowledge, acceptability, and use, as compared with a mass media campaign only.

### Objectives and hypothesis

**Fig. 1:**
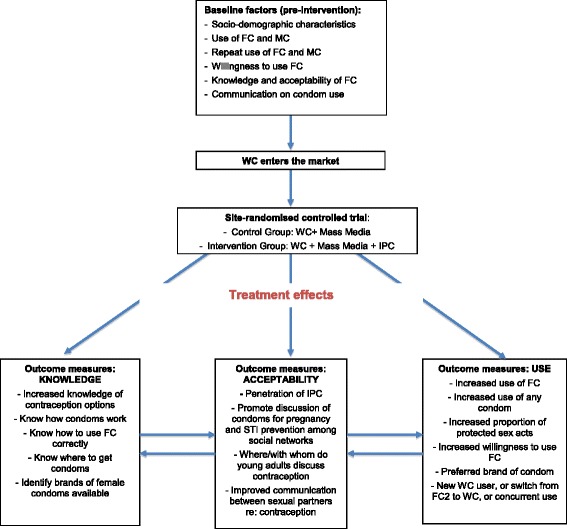
Summary of conceptual framework

The overall objective of this RCT is to evaluate how the introduction of the WC impacts the condom market in Zambia. Specifically, we will evaluate the effectiveness of IPC plus mass media plus distribution of WC (intervention) compared to mass media plus distribution of WC (control) on the prevalence of WC use and prevalence of overall condom use. The conceptual framework is described in Fig. 1.

The primary objectives of this evaluation are:

1. To assess the effect of the introduction of the WC on the overall condom market (self-reported condom use and self-reported use of WC)
2. To determine the additional effect of an IPC intervention on self-reported *use of the WC* as compared with just mass media including a website (controlling for any differences between the intervention and control groups)
3. To determine the additional effect of an IPC intervention on self-reported *use of any condom* as compared with just mass media including a website (controlling for any differences between the intervention and control groups)
4. To determine the additional effect of an IPC intervention on *willingness to use an FC* (and specifically, the WC) as compared with just mass media including a website (controlling for any differences between the intervention and control groups)

The secondary objectives of this evaluation are:

5. To determine the effect of an IPC intervention on self-reported *knowledge, attitudes, and practices regarding the FC, specifically the WC*, as compared with just mass media including a website (controlling for any differences between the intervention and control groups)
6. To determine the effect of an IPC intervention on community-level *acceptability of an FC, specifically the WC*, compared with just mass media including a website (controlling for any differences between the intervention and control groups)
7. To determine the effect of an IPC intervention on self-reported *changes in interpersonal communication with sexual partners* regarding contraceptive use as compared with just mass media including a website (controlling for any differences between the intervention and control groups)
8. To assess women’s and men’s preferences for condom brands.

## Methods/design

The overall aim of this study is to assess the impact of introducing the WC on the overall condom market. We will also evaluate the effectiveness of IPC plus mass media plus distribution of WC (intervention) compared with mass media plus distribution of WC (control) on the self-reported prevalence of WC use and prevalence of overall condom use using an RCT design. Forty urban wards in Lusaka and nearby districts (Chilanga, Kafue, and Chongwe) will be randomized to the control or intervention arm with a 1:1 allocation ratio. The baseline survey conducted in all 40 wards will be analyzed as a quasi-control arm in order to assess the effect of the introduction of the WC on the total condom market. This will be done for two reasons: (1) there are no comparably urban areas in the country to act as control settings; (2) the potential for spillover for a new condom product and its marketing campaign is extremely high, making a true control arm logistically and technically impossible. The media campaign will be displayed on a website and billboards throughout the city; thus these cannot be geographically limited.

When the baseline survey is implemented, no WC will be available, as is the status quo in Lusaka. After rollout of the WC, wards in the control arm will receive only the WC and a mass media campaign. Intervention wards will receive the WC, a mass media campaign, and the IPC intervention. The RCT component of this study will aim to evaluate the added impact of the IPC intervention compared with just distribution of the WC and the mass media campaign. The trial will be evaluated through baseline and endline questionnaires administered to eligible individuals residing within randomly selected households located in these wards. The Standard Protocol Items: Recommendations for Interventional Trials (SPIRIT) checklist was used to prepare the study protocol (Additional file 1). The resulting protocol schedule of procedures and activities for this study is outlined in Table 1 as per the SPIRIT guidelines.

**Table 1:** SPIRIT protocol schedule of procedures and activities for Maximum Diva study

	Study period					
	Prep. phase	Baseline survey	Randomization	Intervention period	Endline survey	Close-out
Activity	*-t*_*1*_	0		*t*_*1*_	*t2*	*t3*
Enrollment:						
Sampling and mapping	X					
Informed consent written	X					
Hiring and training surveyors	X					
Piloting, revising survey tool	X					
Interventions:						
Randomization			X			
Distribution plus mass media (control)				X		
IPC (intervention)				X		
Assessments:						
Baseline survey		X				
Baseline outcomes of interest: male condom use in last 6 months, male condom use at last sexual intercourse, types of contraception used, interest in using female condom in the future		X				
Endline survey					X	
Endline outcomes of interest: WC use (last 6 months, last sexual intercourse), attended IPC, knowledge of WC, interest in using WC					X	
Dissemination:						
Write up manuscripts for peer-reviewed publication, present at relevant international and local conferences, host dissemination event with local stakeholders						X

### Setting and participants

**Fig. 2:**
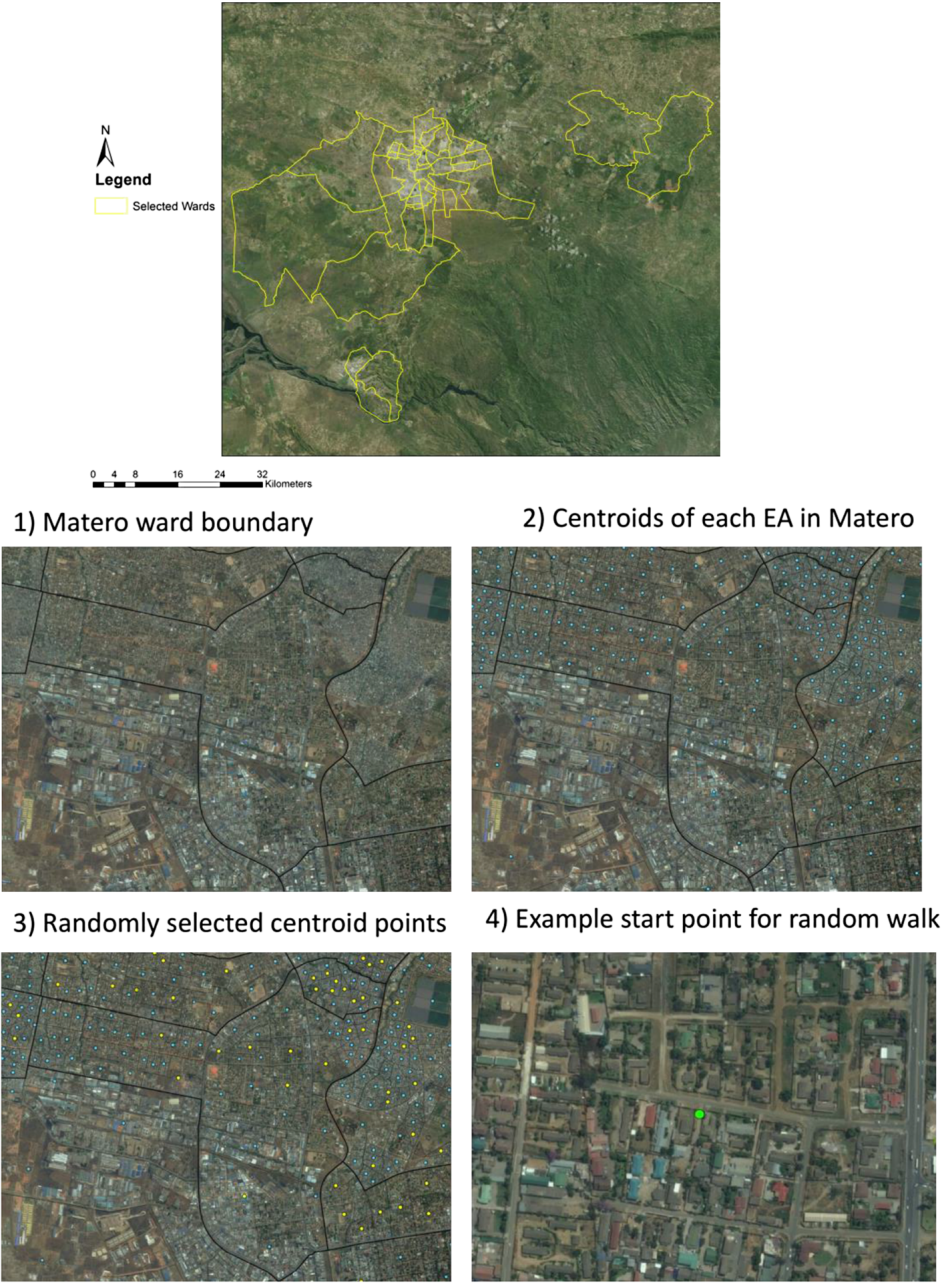
Map of selected wards in Zambia, and sampling methodology (example: Matero ward)

The new WC product is being distributed in urban wards that comprise Lusaka, the capital of Zambia (see map of selected wards; Fig. 2). A baseline survey was conducted in November 2015, and an endline survey will be conducted in March 2017, each sampling from randomly selected households within 40 selected urban wards. One individual per household will be enrolled and administered the questionnaire if the person has lived in the house for at least the past 6 months (to ensure exposure to the WC and the IPC intervention in the intervention wards), is between the ages of 18 and 24 years, and is sexually active (has had sex in the last 6 months) (eligibility criteria). Both men and women will be included in the study.

### Intervention

The WC program comprises three components: (1) distribution of the WC, (2) a mass media campaign, and (3) an IPC intervention. These are defined as follows:

1. Distribution of the WC: The WC will be distributed to pharmacies and other outlets such as chemists and stores by the SFH located in the geographic areas where the study will be implemented. The distribution of the WC will take place across both study arms. Information regarding where the WC is stocked and how often it is available will be collected throughout the study to determine availability of the product and whether that varies across wards.
2. Mass media campaign: The WC will be advertised widely, targeting young adults through radio, billboards, news media, social media, and a mobile website. The mobile website and accompanying media will be branded under the “Smart Choices” campaign (SmartChoices.co.zm). The website will serve as a main venue for the introduction and promotion of the WC. The media campaign in its various forms will disseminate information about family planning and serve as an interactive forum for young men and women to ask sexual health related questions and generally discuss SRH related topics. SMS blasts and social media messaging will be used to direct individuals to the website.
3. Interpersonal communication (IPC): IPC agents will be recruited from wards within the intervention arm. They will be paid and trained by the SFH on communication skills, sexual and reproductive health information, technical specifications and use of the WC, and condom negotiation skills with sex partners. IPC agents will set up an IPC point in a high traffic area(s) of each ward selected for the intervention arm for initial demand creation, and will use their existing and new contacts to access networks of young people in the area for smaller group and one-on-one information sessions. The IPC intervention will comprise communication messages focused on how to use and the benefits of the use of WCs and male condoms for the prevention of unplanned pregnancies. IPC activities will be targeted to both young men and women (aged 18–24 years). The IPC agents will also be young (18–35 years of age) males and females. The key components of the IPC intervention include (1) targeting men and women aged 18–24 years, (2) providing content on the importance of contraceptive use with a focus on the benefits of FCs, (3) demonstrating how to use the WC on a pelvic model, and (4) training on condom use negotiations with a sex partner through theater or role playing. Information will also be distributed in the form of pamphlets, and tools used for the IPC sessions will include flip charts and tablets.

### Sample size

To calculate the sample size needed in each trial arm, we considered prevalence of any condom use (percentage) as our outcome of interest. The contraceptive prevalence rate (CPR) for any modern contraceptive method in urban Zambia (for 15- to 49-year-old married women) is currently about 53% [4]. However, our study includes both married and unmarried women. A report from 2009 by the Zambian Statistics Office reported urban condom use to be 54% in Zambia [7]. We used 53% to calculate our sample size, and aim to detect a minimum increase of 5% use with 80% power at the 95% significance level. Because the trial is clustered at the ward level (with many individuals living within a ward), the sample size needs to be increased to account for intraclass correlation (ICC) within wards. However, no information was found from previous studies on the likely between- or within-cluster correlations in FC use in previous cluster-randomized trials. When the ICC cannot be estimated, common practice is to double the sample size to account for clustering.

Using Stata (StataCorp, College Station, TX, USA), we calculated the sample size required to detect a 4%, 5%, and 7% increase in any use from a 53% baseline use and an estimated standard deviation (SD) of 0.15, doubling the sample size to account for the unknown ICC. Lastly, we added 10% to increase the sample size to account for potential refusals. We will use a sample size of 1157 individuals per arm (a total of 2314 individuals in both arms). This yields a total of 58 individuals to be interviewed per ward, in a total of 40 wards (see Table 2).

**Table 2:** Sample size required to detect a 2, 3, and 4 percentage point increase in condom use (4%, 5%, and 7% increase)

Hypothesized standard deviation of condom use	Hypothesized increase in proportion of condom use between groups	Sample size required in each group (ratio 1:1)	Adjusted for effect of clustering (design effect = 2)	10% refusal rate (estimated)
0.15	7%	296	592	651
0.15	5%	526	1052	1157
0.15	4%	1183	2366	2603

### Survey and randomization

The enrollment of participants for the baseline survey was based upon a systematic random sampling strategy: (1) selection of wards, (2) household sampling, and (3) individual sampling. These Wards were selected because they are urban/peri-urban and comprise Lusaka, the main city of Zambia. In order to select households, all 40 wards were subdivided into enumeration areas (EAs) based on household density. The centroid point of each EA was generated using ArcGIS version 10.2 software (Esri, Redlands, CA, USA). Centroid points that are within 150 m of the ward boundary were excluded, to avoid enumerators approaching houses that are not in the selected ward. Five EA centroid points and five back-up points will be randomly selected per ward. Enumerator supervisors visited each GPS point and directed enumerators into a Random Walk from each selected EA centroid point, visiting every other house in each direction. A map of Lusaka wards and an example of the sampling strategy are detailed in Fig. 2. Interviewers visit households in the mornings and afternoons, and on weekends, when participants are likely to be home. When the enumerator visits a selected household, he/she records a roster of individuals living in that house. The roster assigns each individual a number, then one individual is randomly selected for participation. If that participant is not eligible, the next randomly selected individual from the roster will be approached. If no one is eligible, then the house will be labeled “no one eligible.” If the participant is home but busy, the interviewer will schedule an appointment for a later time. If no one is home, the interviewer will approach one additional time. If no one is home at the second visit, then a GPS coordinate is recorded and the household labeled “no one available.” If the individual at the household refuses to participate, then the household will be labeled “refused.”

A structured questionnaire was administered to each eligible and consented individual who met the inclusion criteria. Survey interviewers were either male or female and work for Innovations for Poverty Action (IPA) Zambia. Each interview lasted about one hour and took place in the home but away from other household members, in private. The questionnaire collects socio-demographic information, information regarding condom use, sexual behavior, knowledge and beliefs specifically regarding condoms, contraceptive options, and communication with sexual partners. A small reimbursement amount was provided to participants who completed the survey. The study enrolled both men and women.

After the baseline survey was completed in January 2016, all 40 wards were randomized to receive the control (WC plus mass media) or intervention (WC plus mass media plus IPC), and were allocated by an IPA statistician using a Stata program. Ward level population density based on the 2010 census data, ward level poverty, and average distance to the nearest health facility were used to stratify the randomization. Additional balance checks were conducted to ensure randomization was successful. Each ward has been mapped including the IPC sites, and all visited houses enumerated and given an ID. Since randomization, the IPC intervention has been implemented in intervention wards only. Only the IPA statistician and the implementing partner (the SFH) know the allocation of wards. The endline survey is planned for March 2017, and will follow the same protocol as outlined above for the baseline survey. The endline survey will have additional questions regarding exposure to the mass media and IPC intervention, including pictures to prompt recall. Households will be randomly selected for both surveys; the same households will not be approached.

**Fig. 3:**
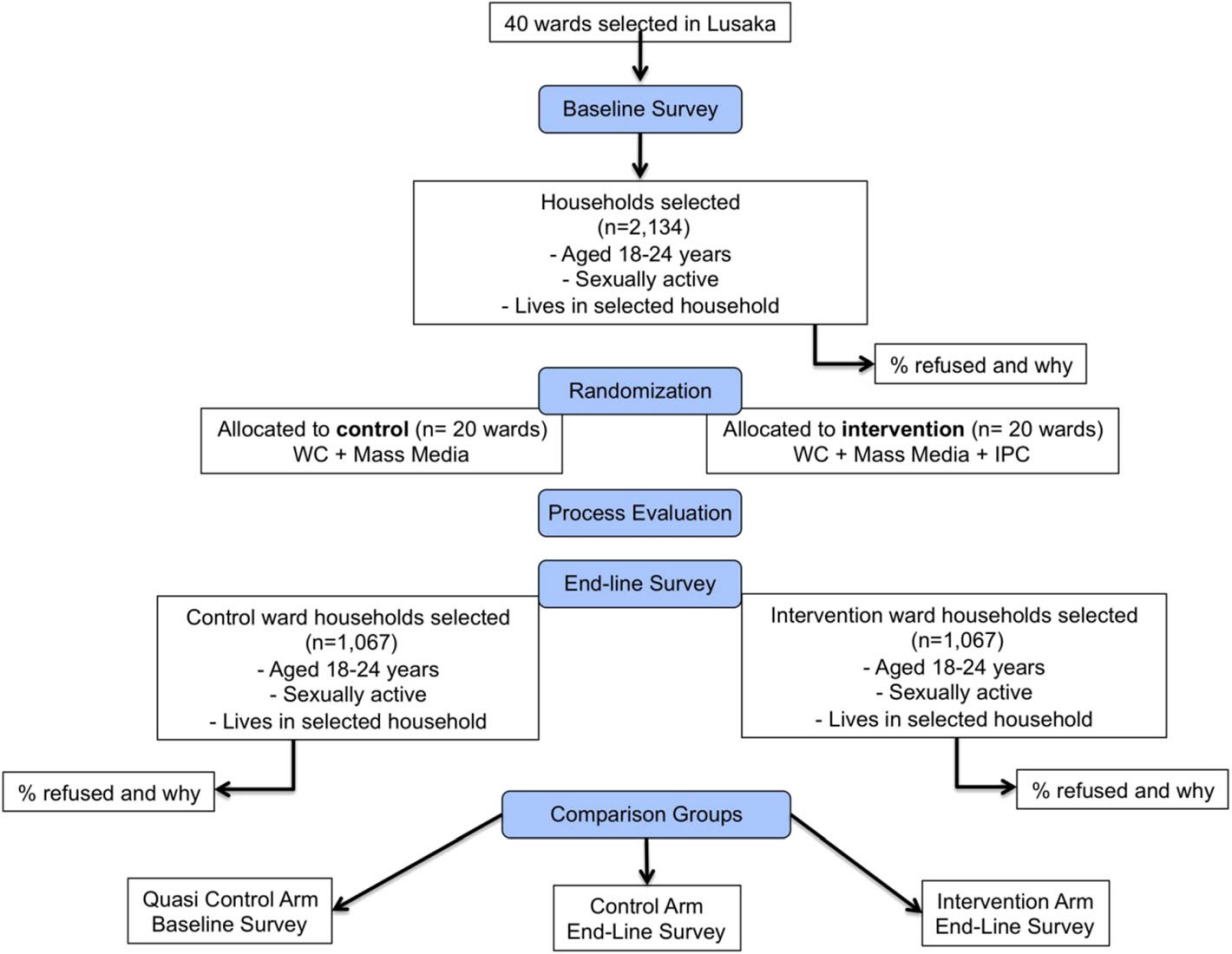
CONSORT flow diagram of study design

The Consolidated Standards of Reporting Trials (CONSORT) randomization and survey structure are described in Fig. 3.

### Primary and secondary outcomes

The primary and secondary outcomes listed below correspond to the primary and secondary objectives.

The primary outcomes are:

1. Increase in the *prevalence (percentage) of WC* (self-reported) use among men and women aged 18–24 years ever
  - ever
  - last 6 months,
  - most recent sexual intercourse.

2. Increase in the *prevalence (percentage) of any condom* (self-reported) use among men and women aged 18–24 years
  - ever
  - in the past 6 months
  - at most recent sexual intercourse.

3. Increase in the *willingness to use* the WC in the future among men and women aged 18–24 years.

The secondary outcomes are:

4. Self-reported increase in knowledge, improved attitudes, and practices of both male and female condoms after attending IPC intervention
5. Self-reported increase in acceptability (community awareness) of FCs
6. Self-reported improvement in communication with sexual partner(s) regarding use of male and/or female condoms for STI prevention and/or family planning
7. Self-reported condom brand preference before and after rollout of the WC.

### Process evaluation

The RCT will be complemented by a process evaluation that is being conducted between the baseline and endline data collections. After the baseline survey was conducted, the intervention was rolled out: the WC is now be available throughout Lusaka coupled with the mass media campaign. The IPC intervention is being implemented in the randomly selected intervention wards. Since completion of baseline, IPA and SFH have been carrying out a process evaluation to assess the fidelity of the various components of the WC project as well as penetration of the IPC intervention. This is being done to assess the following: (1) availability and distribution of the WC, (2) tracking of the mass media campaign, (3) penetration and reach of the IPC intervention, and (4) acceptability of the WC.

First, to monitor the availability and distribution of the WC, we will review monitoring data from SFH to track the availability of the WC throughout Lusaka. SFH is keeping track of distributions of the WC to determine how many WC products are purchased throughout the year. To assess availability to potential customers, we are implementing mystery shopper techniques to visit pharmacies and other distribution venues in all wards to determine if the WC is available and in stock. This can be cross-compared with records kept by the SFH of how many WCs have been purchased and distributed throughout Lusaka.

Second, we are working with SFH to ensure that all components of the mass media intervention are consistently rolled out, for example, checking that the website is up to date and the pages are successfully loading. The billboards are being checked periodically to ensure that they are still being viewed.

Third, we are working with SFH to randomly visit IPC recruitment and sessions. We are conducting “spot checks” and attending a random selection of IPC activities to determine if the IPC is being implemented and if the IPC agent is adhering to the training curriculum and key components of the intervention. We are also comparing logs with IPC agents regarding how many individuals attend the IPC. IPC agents are collecting ”client forms” during IPC sessions to provide information on the number of participants, date, location, and gender composition of the sessions. We have developed and implemented monitoring tools to collect information at the IPC sessions on participation, quality of delivery, and frequency of IPC sessions in each ward to measure the intervention intensity and penetration. The evaluation team is also monitoring the process of setting up IPC appointments and tracking (on random visits) how many invitations for IPC are sent out to measure take-up of the intervention. This is running parallel to the administrative/monitoring data that SFH is collecting.

Lastly, 30 qualitative focus group discussions are being conducted to understand how the WC product is being received in the communities. SFH may also collect short user surveys or collect qualitative data that can be shared. This will help determine if the product is available and if the mass media is being seen/accessed. It will also help us understand any reactions to the WC, how the messaging is being interpreted, and the acceptability of the new product to the community.

The evaluation team will work closely with the SFH team to ensure all administrative data needed for analysis are collected during the intervention. We will also note if any other major SRH activities are occurring throughout the study period, for example, any other products added to the market, any major stockouts of existing products, or any widespread education campaigns that might impact the IPC intervention. Overall this process evaluation will determine key factors that may impact the outcomes of the IPC intervention (i.e., availability of the WC, the consistency of the media campaign, and the quality of and reach of the IPC intervention). The process evaluation began when the project was rolled out, and is ongoing until the endline survey begins.

### Data management and analysis plan

The data are collected into an Android phone using SurveyCTO software; the data obtained are cleaned and exported into Stata for analysis. A high frequency check is run every two to three days to highlight any errors in data collection. Twenty percent of all participants (eligible or ineligible) are being randomly selected for back checking. The back check includes confirmation of ineligibility or administration of a 5- to 10-min version of the original survey to compare responses. All data are encrypted using Boxcryptor and stored on the SurveyCTO cloud and Box. A de-identified version of the data and statistical code will be shared on Dataverse at the completion of the study.

We will report the trial according to the CONSORT standards for reporting RCTs. This is a behavioral intervention unlikely to produce adverse effects, so analysis will be undertaken immediately once the endline survey has been completed. Baseline survey data will be analyzed to measure differences between the groups and between the wards at baseline. Randomization can be checked at this point. The main analysis will take place after endline data have been collected, to measure the impact of the introduction of the WC and the IPC intervention on condom use, controlling for any potential confounders or explanatory factors detected from the baseline analysis.

To assess the impact of the introduction of the new and premium WC on the Zambian condom market, the evaluation team will use the baseline survey as a quasi-control arm. While there are limitations with this approach, it will allow for measurement of changes in condom use, brand switching, user preferences, and uptake of the new product. The main limitation is that the study cannot determine attribution; however, given the challenges to create a better counterfactual, the evaluating and implementing teams agreed that this is the best approach in view of programmatic constraints.

The baseline survey will occurred at a time when there was no WC product available, no mass media, and no IPC. The study outcomes are self-reported condom (male or female) use ever, during the past 6 months, and at last sexual intercourse. The study will measure these indicators at baseline (no WC available) and endline (WC available). By analyzing changes in these indicators, the study will show changes in overall condom use as well as uptake of the WC in areas with only mass media versus mass media and IPC.

For the RCT component of the study, we will analyze the impact of the IPC intervention on our outcomes of interest (male condom use, WC use, willingness to use the WC), comparing intervention to control wards. Analyses will be conducted in Stata v12 (StataCorp, College Station, TX, USA). Results will be adjusted for any ward level clustering if it is identified. Multivariate regression analyses will be carried out to determine the impact of the IPC intervention on condom use (male condom and WC), controlling for demographic characteristics of participants.

#### Statistical methods

For the primary and secondary outcomes we will estimate odds ratios with 95% confidence intervals. We will calculate the rates of self-reported contraception use per ward and willingness to use the WC. Descriptive analyses will be conducted, and several regression models will be run to determine the impact of IPC on each outcome variable of interest. All regression models will control for baseline characteristics of relevance to the outcome (either as potential confounders or as explanatory factors). Backward elimination methods will be used to remove non-statistically significant factors that do not improve the fit of the model and do not confound other associations observed. Multilevel models may be implemented to control for individual and ward level correlation, as individuals are nested within wards.

### Ethics

Individuals who meet the study inclusion criteria will give written informed consent. The interviewer will inform the participant that a study is being conducted on contraception, specifically knowledge and use of FCs, in order to improve SRH outcomes for this community, and that their time is very much appreciated and valued. The participant will receive an information sheet with additional details regarding the study in which they are enrolling. After this step, the participant will receive and sign for the small compensation (30 kwacha of talk time), and the questionnaire can be administered. Interviewers will conduct the survey in a private area to protect anonymity and reduce the likelihood that responses are influenced by external household factors.

The questionnaires will be assigned an anonymous ID number, and de-identified. They will be stored safely and not accessed by anyone except the evaluation team (principal investigators, co-investigators, and analysts), for confidentiality. All personal identifying information (PII) will be removed from the dataset. This study has received approval from both IPA’s and Zambia’s institutional review boards, IRB (IRB No. 10854) and Excellence in Research Ethics and Science (ERES) Converge (IRB No. 00005948). Any modifications to the protocol or survey will be submitted to both IRBs for review but are not anticipated. We have also received Zambia’s Ministry of Health’s approval to implement the study. The informed consent form and information sheet are provided in Additional file 2.

## Discussion

Results from the trial will provide rigorous evidence on the impact of introducing the new WC product on overall condom use and effective strategies to increase knowledge, acceptability, and uptake. This trial will contribute towards the evidence base on social marketing of condoms and community-based IPC interventions to increase uptake. The focus on young adults in urban areas and their knowledge and use of contraceptives has not been well studied. This trial is aimed at understanding the introduction of a novel contraceptive product to urban youths, and is unique in a number of ways.

First, this is the only RCT of the newly designed and marketed WC in urban sub-Saharan Africa. No studies have evaluated the impact of introducing a premium FC product (the WC) in a market where male condoms and the FC2 have been available. Further, an impact evaluation of social marketing comprised of mass media and an IPC intervention to increase condom use among a general population of young adults has never been conducted, although smaller studies between 1990 and 2010 suggest that social marketing can increase condom use [8]. One recent cluster RCT measured the impact of a Facebook page on the use of condoms for STI prevention and found a positive effect, but noted that it diminished over time [8]. Studies suggest that, despite their widespread availability and low cost, male condoms are not used as often or as consistently as they should be; therefore, adding the WC to the market could increase the levels of protected sex overall [9]. Studies suggest that when both types of condoms are available, consistent users will alternate male and female condoms as complements, not replacements for each other [9]. The WC may also be used more by long-term couples, whereas male condom use decreases significantly once relationships are defined [10]. The WC may serve as an alternative for women who are not able to negotiate male condom use [10].

Second, this is the first RCT of a community-based IPC intervention led by paid, trained IPC agents and targeting the general youth population. Some recent studies have highlighted aspects of IPC that can make it more successful, such as inclusion of stories and dialogue, demonstrations, role playing, games, and negotiation skills. IPC interventions that are dialogue-based are well suited for promoting openness and discussion about sensitive or stigmatizing topics such as condom use among couples and peer networks. Social networks can be used to diffuse information about condoms and reinforce condom use norms through IPC, increasing acceptability [11]. Previous programs or interventions with messages highlighting the positive benefits of the FC (e.g., increased sexual pleasure and autonomy) may also increase uptake [3]. IPC interventions that include demonstrations can be critical in improving women’s comfort level with FCs and shift attitudes towards the FC [12]. Women report that the overall acceptability of the FC improves over time with practice [13–15]. A US trial found the strongest predictors of FC use were having had instruction and skills training, and intending to use the method [16]. Knowing how to use an FC and feeling comfortable with insertion is critical, and initial findings suggest that demonstration is a necessary component for a successful rollout.

No single trial or initiative will answer the question of how the WC specifically will impact the condom market, and whether uptake will increase with mass marketing and IPC intervention. This trial will provide evidence on effectiveness of IPC interventions for increasing knowledge, acceptability, and uptake of the WC and condoms, generally. The generalizability of these findings may depend on the marketing of the WC and the culture in other regions of Zambia or in a different region. For example, the promotion of the FC as a woman-controlled method backfired in some countries because male partners viewed FCs as threatening to their control over their partner’s sexual behavior [17, 18].

Recommendations on the impact of IPC approaches for increasing condom uptake and other SRH services will be developed based on the results of this trial. Findings will be written into a report for PSI and WomanCare Global, and a paper will be submitted for publication to a peer-reviewed journal. The evaluation team will work closely with the SFH and PSI to organize a dissemination event subsequent to the completion of the study to share results with local and global stakeholders (government officials, non-governmental organizations (NGOs), international NGOs (INGOs), donors) working on SRH research, programs, and policies in Zambia and at the global level.

## Trial status

At the time of submission, the trial is completing the intervention period and planning an endline cross-sectional survey for January 2017.

## Additional files

Additional file 1: SPIRIT 2013 Checklist: Items addressed for the Maximum Diva Woman’s Condom Randomized Controlled Trial. (DOC 123 kb)
Additional file 2: Informed consent form and participant information sheet (English) for the Maximum Diva Woman’s Condom Randomized Controlled Trial. (DOCX 18 kb)

## Abbreviations

CPR: Contraceptive prevalence rate
EA: Enumeration area
EECO: Expanding Effective Contraceptive Options
FC: Female condom
FP: Family planning
HIV: Human immunodeficiency virus
ICC: Intraclass correlation
IPA: Innovations for Poverty Action
IPC: Interpersonal communication
IRB: Institutional Review Board
PII: Personal identifying information
PSI: Population Services International
RCT: Randomized controlled trial
SFH: Society for Family Health
SPIRIT: Standard Protocol Items: Recommendations for Interventional Trials
STI: Sexually transmitted infection
WC: Woman’s Condom
