# Supplementary material for: Impact of communication strategies to increase knowledge, acceptability, and uptake of a new Woman’s Condom in urban Lusaka, Zambia: study protocol for a randomized controlled trial
Source: Trials. 2016 Dec 13;17:596. doi: 10.1186/s13063-016-1681-x (PMC5154147; doi:10.1186/s13063-016-1681-x)
Supplement: Additional file 2: — Informed consent form and participant information sheet (English) for the Maximum Diva Woman’s Condom Randomized Controlled Trial. (DOCX 18 kb) [file 13063_2016_1681_MOESM2_ESM.docx]

Additional file 2: Informed consent form and participant information sheet (English) for the Maximum Diva Woman’s Condom Randomized Controlled Trial

**CONSENT FORM**

**TITLE OF RESEARCH: The Impact of an Interpersonal Communication Intervention on Uptake of the Maximum Diva Woman’s Condom in Lusaka, Zambia**

**REFERENCE TO PARTICIPANT INFORMATION SHEET:**

1. Make sure that you read the Information Sheet carefully, or that it has been explained to you to your satisfaction.

2. Note that no taping or ‘audio’ recording will be used.

3. Your participation in this research is entirely voluntary, i.e. you do not have to participate if you do not wish to.

4. Refusal to take part will involve no penalty or loss of services to which you are otherwise entitled.

5. If you decide to take part, you are still free to withdraw at any time without penalty or loss of services and without

giving a reason for your withdrawal.

6. You may choose not to answer particular questions that are asked in the study. If there is anything that you would prefer not to discuss, please feel free to say so.

7. The information collected in this interview will be kept strictly confidential.

8. If you choose to participate in this research study, your signed consent is required below before I proceed with the interview with you.

---------------------------------------------------------------------------------------------------------------------------------------------------------------------

**VOLUNTARY CONSENT**

I have read (or have had explained to me) the information about this research as contained in the Participant Information Sheet. I have had the opportunity to ask questions about it and any questions I have asked have been answered to my satisfaction.

I now consent voluntarily to be a participant in this project and understand that I have the right to end the interview at any time, and to choose not to answer particular questions that are asked in the study.

My signature below says that I am willing to participate in this research:

Participant’s name (Printed): ………………………………………..............……………………………………………………………....

Participant’s signature or thumbprint: …………………………………………………… Consent Date: ………………………...............................

Researcher or Intervierwer Conducting Informed Consent (Printed) ………………………………………………………………….………………….

Signature of Researcher: ……………………………………………………………….………….. Date: …………………………………………………………………

**INNOVATIONS FOR POVERTY ACTION**

**PARTICIPANT INFORMATION SHEET**

**TITLE OF RESEARCH:** The Impact of an Interpersonal Communication Intervention on Uptake of the Maximum Diva Woman’s Condom in Lusaka, Zambia

**PURPOSE OF THE STUDY:** A third generation female condom called the Maximum Diva Woman’s condom will be marketed in Lusaka, Zambia. The Maximum Diva Woman’s Condom is a new type of female condom that provides non-hormonal protection against unintended pregnancy and sexually transmitted infections. This study will evaluate the effect of introducing a new female condom to the Zambian market, a mass media campaign, and interpersonal communication intervention on the prevalence of female condom use and prevalence of overall condom use.

Specifically, the study will examine the effect of these two approaches on female and male condom use. To do this, we ask questions about your knowledge, attitudes and practice regarding female and male condoms. To understand the effect of interpersonal communication, the proposed study will ask you questions on communication with sexual partners. The results of this study will be used to inform policy makers and practitioners in Zambia about how to design future programs aimed at improving contraceptive use.

**DESCRIPTION OF THE STUDY AND YOUR INVOLVEMENT:** You will be one of approximately 2,314 individuals to be asked to participate in this project in Lusaka Province. If you are willing to participate in this study, we will ask you a few questions about sexual behaviour and contraceptive use – the interview will occur just outside your home and take approximately 30 – 45 minutes. No recordings will be made of the interview. The researcher will ask all questions in the language you prefer – Bemba, English, or Nyanja. The study is being funded by Population Services International (PSI) and is being conducted by Innovations for Poverty Action (IPA), an international nonprofit organization that creates and evaluates solutions to social and development problems.

**CONFIDENTIALITY:** Your interview responses and any other information that you provide to the study will be treated with confidence. This means that we will not use your name, or that of anyone you mention, in any discussions of the research or in any reports that come out of the project. Any information that identifies you will be separated from your other answers so that only the researchers involved in this study will be able to track your responses back to you. The surveys will be kept in the Innovation for Poverty Action office premises in a locked cupboard/filing cabinet for 5 years, at which point they will be destroyed.

**VOLUNTARY PARTICIPATION AND WITHDRAWAL:** Your participation in this study is completely voluntary. If you do not want to participate or wish to withdraw from the study, you can do so at any time. If you choose to participate, you are not obligated to respond to all survey questions. You can skip any question or section that you do not wish to respond to. Your participation is voluntary at any stage and you are free to withdraw from any activities that you do not wish to complete without any negative repercussion.

**RISKS:** The study presents minimal to no risk to you and to other members of your household. As described, all participation is voluntary and all participant information will remain confidential.

**BENEFITS:**

There will be no direct benefit for your participation in this study but the findings will be used to help design better health policies and programs for other Zambians. You will receive 30 kwacha of talk time at the end of the survey to thank you for your time.

**CONTACTS FOR QUESTIONS**

For more information or if you are unhappy or if you have a complaint concerning the manner in which this research, you may contact the following people:

1. Rachna Nag Chowdhuri, Co-Investigator, Innovations for Poverty Action. Physical address: Plot 32, Mwambula Road, Off Central Street, Jesmondine, Lusaka. Mailing address: Post.Net Box 653, P/bag E891, Manda Hill, Lusaka, Zambia. Telephone: 0971 775 754, Email: [rnchowdhuri@poverty-action.org](mailto:rnchowdhuri@poverty-action.org)

2. Grace Msichili, Survey Coordinator, Innovations for Poverty Action. Physical address: Plot 32, Mwambula Road, Off Central Street, Jesmondine, Lusaka. Mailing address: Post.Net Box 653, P/bag E891, Manda Hill, Lusaka, Zambia. Telephone: 0977822527 Email: gmschili@poverty-action.org

3. ERES CONVERGE Ethical Review Board, 33 Joseph Mwilwa Road, Rhodes Park, Lusaka, Zambia. Telephone: 0955 155 633. Email: [eresconverage@yahoo.co.uk](mailto:eresconverage@yahoo.co.uk)

4. Innovations for Poverty Action Institutional Review Board. 101 Whitney Ave, New Haven, CT 06510, USA. Telephone: +1(203) 772-2216, Email: humansubjects@poverty-action.org
